# A novel approach to treating mirror hand deformity: a case report of functional thumb reconstruction using a palmar incision design

**DOI:** 10.3389/fsurg.2025.1720877

**Published:** 2025-11-18

**Authors:** Yufeng Shi, Ruibin Hu, Hangchong Shen, Xin Wang, Danya Zhou

**Affiliations:** Department of Hand Microsurgery and Plastic Reconstructive Surgery, Ningbo No. 6 Hospital, Ningbo, China

**Keywords:** case report, mirror hand, treating, ulnar dimelia, clinical features

## Abstract

Congenital anomalies of the hand and forearm present a complex challenge in plastic and pediatric surgery. We present the case of a 13-month-old child with a congenital left mirror deformity, characterized by the presence of eight digits and two ulnae exhibiting near-perfect bilateral symmetry. A novel palmar-based incision design was employed for the corrective and functional reconstruction of the left hand, which may serve as a reference for the surgical management of similar conditions. Preoperatively, the radial-side middle finger demonstrated palmar opposition against the ulnar aspect of the palm. The surgical procedure included resection of the radial-sided index, ring, and little fingers, along with pollicization of the middle finger. Functional reconstruction of the neothumb involved restoring adduction and abduction using preserved native anatomical structures. Following comprehensive clinical and imaging evaluations, the patient underwent successful surgical treatment. Postoperatively, substantial improvements were observed in both hand morphology and grasping function. Through our novel palmar incision approach, we effectively reconstructed thumb opposition and adduction while preserving intrinsic hand musculature and achieving optimal scar concealment. In managing mirror hand deformities, meticulous selection of the most dominant digit for thumb reconstruction remains crucial. At the initial surgical stage, osteotomy of the selected digit was performed to enhance its morphological similarity to a normal thumb.

## 1 Introduction

Mirror hand deformity, first described by Jackson in 1853 (1), is a rare congenital deformity of the hand characterized by the absence of the thumb and a mirror-image duplication of the fingers. A deep central cleft typically separates the ulnar and radial digits.

Mirror hand deformities are classified into two main types according to the anatomy of the forearm bones: 1. Classic type (also known as ulnar dimelia), in which the forearm contains two ulnae; and 2. non-classic type, in which the forearm consists of a radius, along with one or two ulnae (2). The formation of the radioulnar axis during limb development is regulated by a group of mesenchymal cells called the polarized active regions (3, 4). A failure in the normal development of the radius may contribute to the duplication and differentiation of the ulna into two structures (5). Polydactyly affects both hand appearance and function. The duplication of the ulnae results in the absence of the radioulnar joint, limiting forearm rotation. Stiff elbows and polydactyly of the hands are the primary functional challenges associated with this anomaly, often requiring surgical intervention (6).

Traditional surgical approaches to mirror hand deformity include Pollicization of a radial digit (7, 8), Simple excision of the radial polydactyly (9), Index finger pollicization combined with excision of the supernumerary digit (10), and The two-stage rotational osteotomy of the preserved first digit.^2^However, the existing literature lacks focused investigation into how postoperative scar appearance affects psychological development and social well-being. Our technique introduces a novel volar incision design that not only improves thumb appearance and function but also creates a well-concealed scar, which is virtually undetectable from the dorsal view. This superior cosmetic outcome minimizes potential impacts on the child's psychological development and future social interactions, thereby significantly enhancing parental acceptance.We preserved the metacarpal base of the mirror index finger, which maintains the stability of the carpometacarpal joint while providing a bony structure to widen the first web space.

## 2 Case description

Here, we present a case of congenital malformation of the left hand and forearm in a 13-month-old male child, which was successfully treated with surgery.

**Figure 1:**
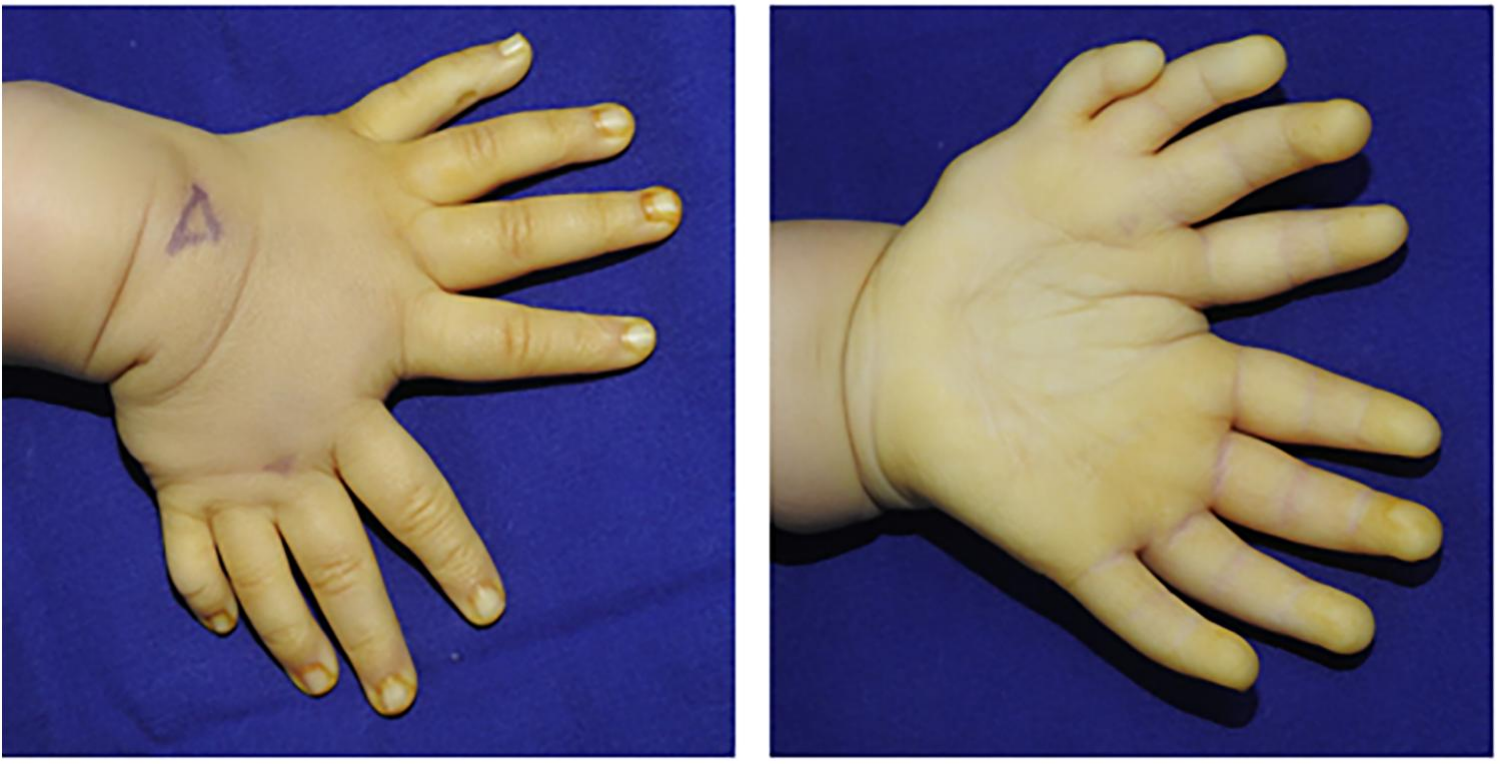
External view of the child's left hand.

The patient was born with a malformed left hand, exhibiting mirror-image polydactyly and a significantly broadened forearm. His body weight and physical development were otherwise normal. He was the fourth child of healthy, non-consanguineous parents, with no family history of genetic disorders over two generations (Figure 1).

**Figure 2:**
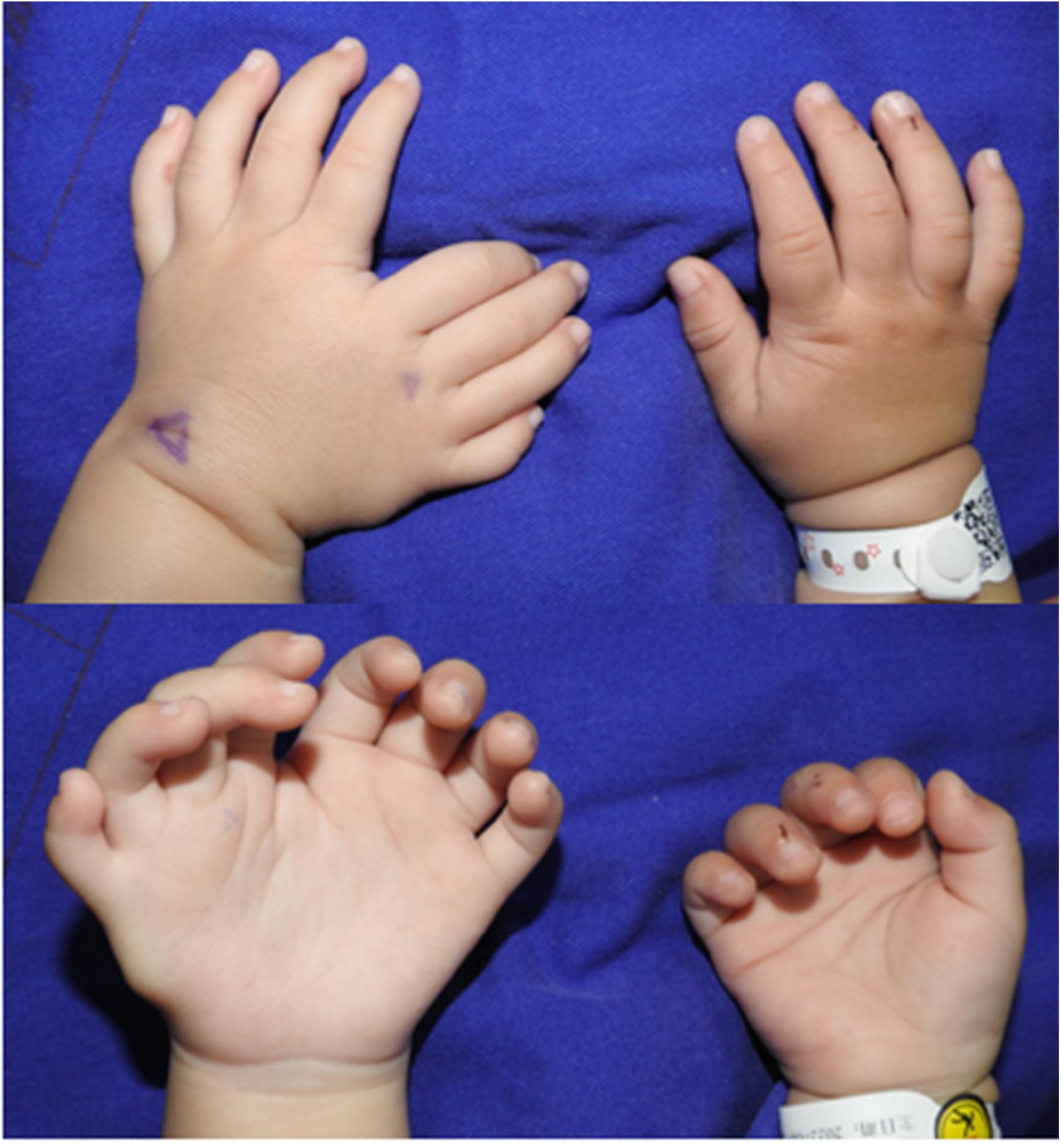
External view of the child's left and right hands.

Physical examination revealed a classic mirror hand deformity. The left hand presented with eight digits arranged in a fan-shaped configuration, exhibiting ulnar polydactyly, thumb aplasia, and a broadened palm. The radial and ulnar components were symmetrical, each comprising four digits and palmar tissue, separated by a deep cleft between the respective index fingers. Functionally, active and passive range of motion was well-preserved in the four ulnar digits. In contrast, the ring and little fingers on the radial side had limited flexion and extension. While the radial index and middle fingers retained flexion capability, their extensor strength was notably weaker than their ulnar counterparts. During active grasp, opposition between the radial middle finger and the ulnar palm was effective. Capillary refill was normal at all fingertips, though reliable sensory assessment was precluded by the patient's age. Active wrist movement was limited due to poor cooperation; passive range of motion was approximately 90° in flexion and 5° in extension. The left forearm was approximately 1 cm shorter than the right, with significant limitations in passive elbow flexion, pronation, and supination (Figure 2). For clarity, the digits on the radial side are hereafter designated as the mirror fingers.

**Figure 3:**
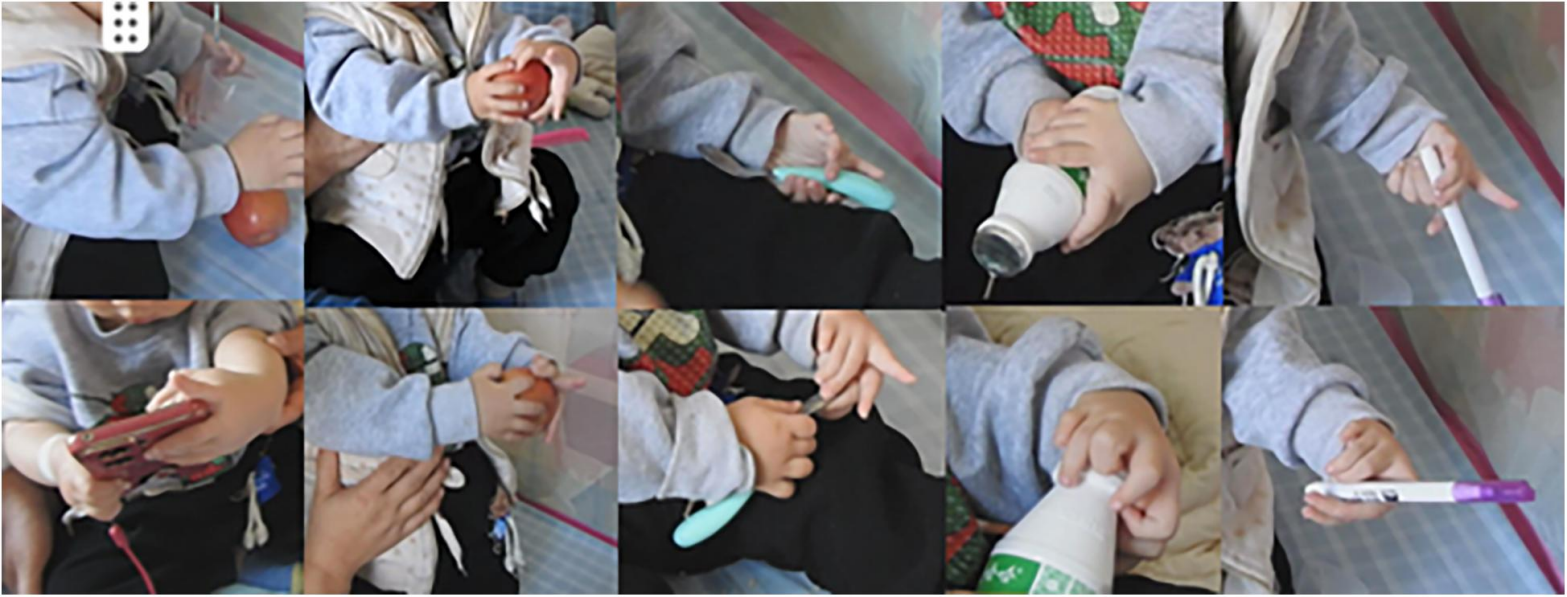
Preoperative photographs showing hand habits and functions.

**Figure 4:**
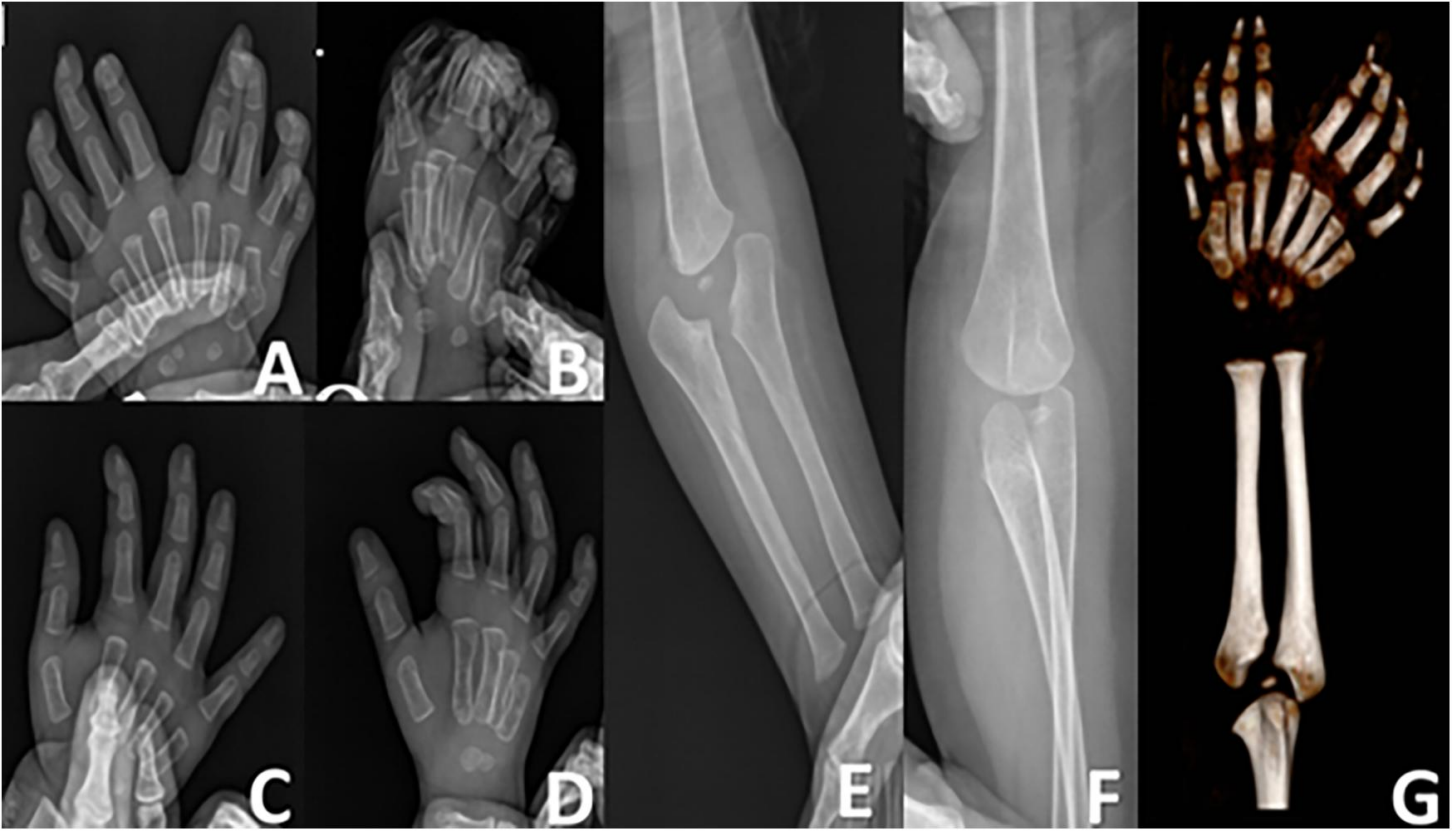
**(A,B)** orthogonal and oblique radiographs of the left hand. **(C,D)** Orthogonal and oblique radiographs of the right hand. **(E,F)** Radiographs of the left elbow joint. **(G)** Three-dimensional computed tomography reconstruction of the left forearm and hand.

The patient had not received any prior treatment or intervention. We documented the patient's preoperative left-hand usage in daily activities to understand their functional habits and to establish a baseline for comparing postoperative improvements in the appearance and function of the left hand (Figure 3). Preoperative radiographic examinations of the left hand, right hand, and left elbow were performed, along with three-dimensional CT reconstruction of the left hand and forearm. These imaging studies provided detailed anatomical understanding of the affected limb and facilitated precise surgical planning (Figure 4). The classification system proposed by Al-Qattan et al. in 1998 (5) is widely used in clinical practice. This patient was classified as type 1A.

**Figure 5:**
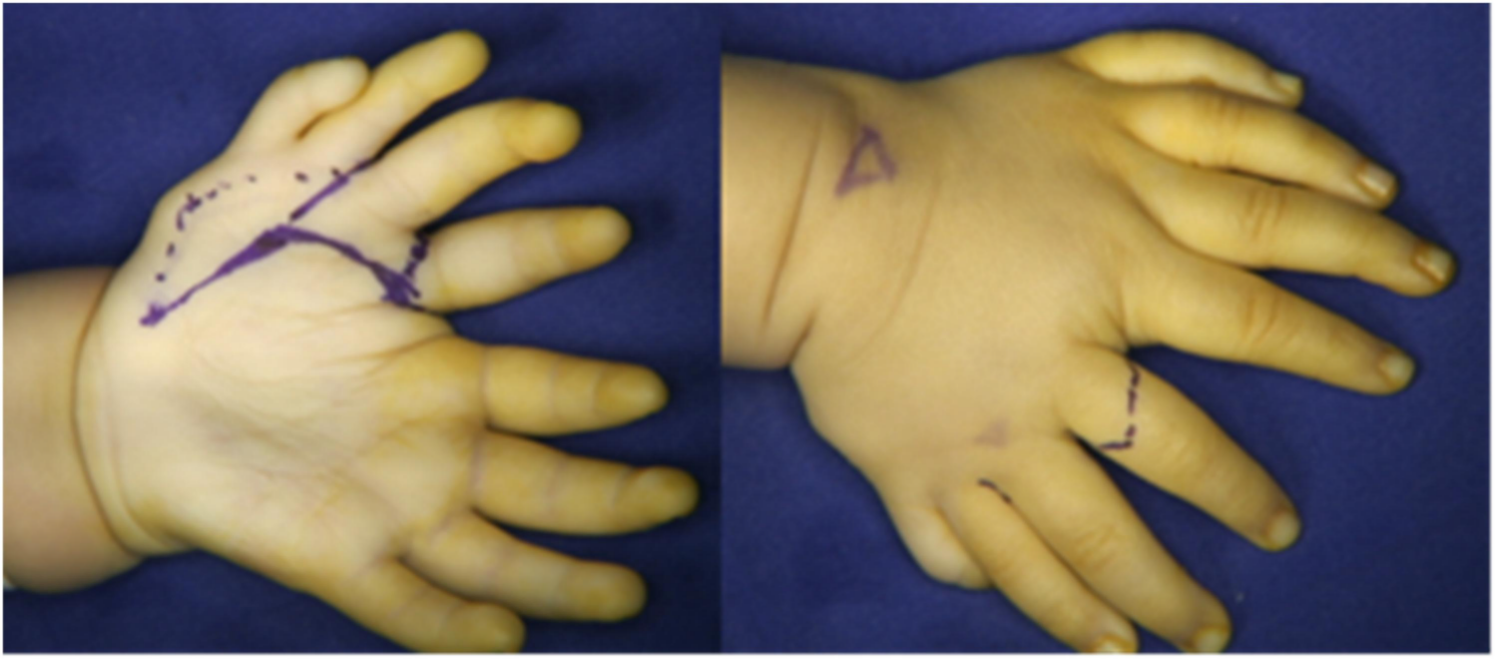
Preoperative incision design.

**Figure 6:**
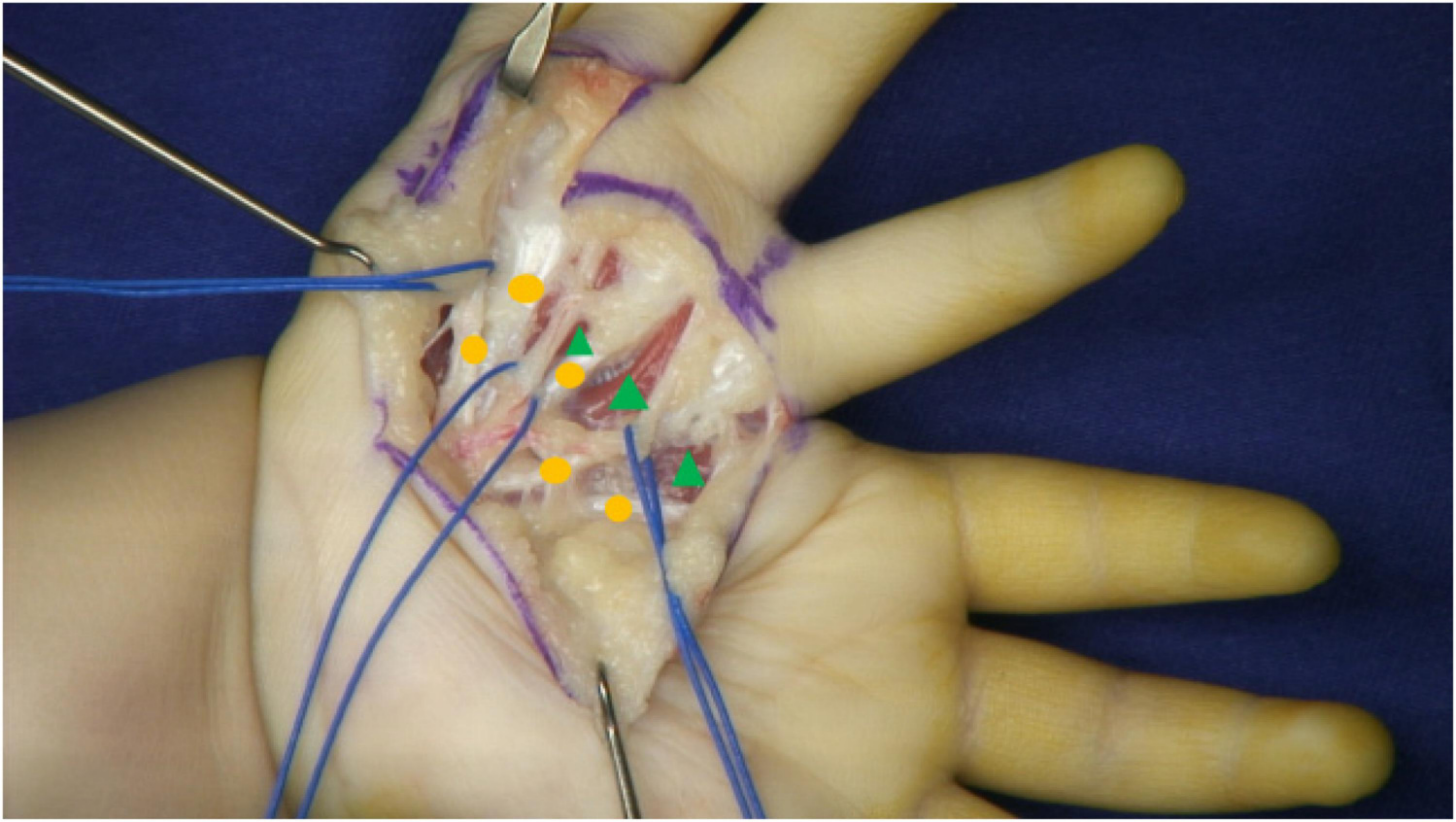
Intraoperative exploration of neurovascular bundles(blue lines), flexor tendons(orange circle), and lumbrical muscles(green triangle).

**Figure 7:**
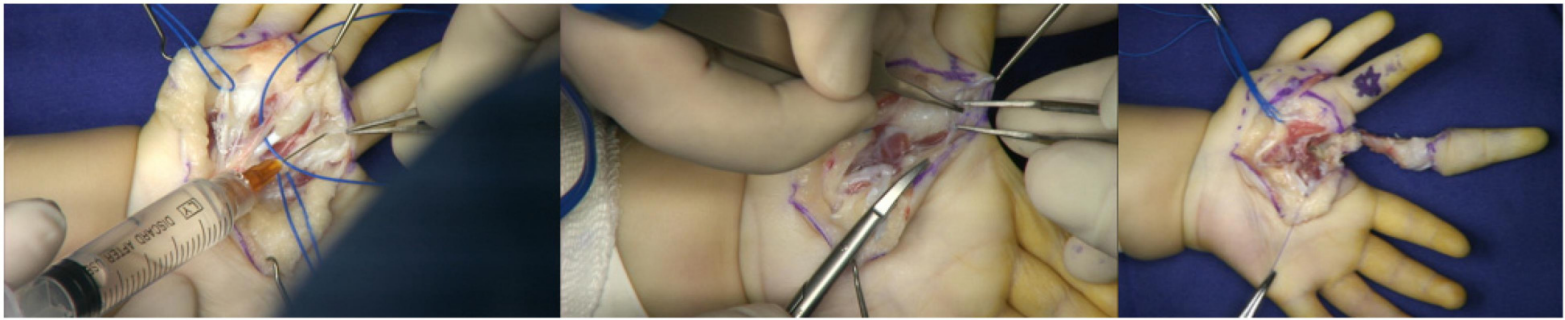
Excision of the mirror index finger.

**Figure 8:**
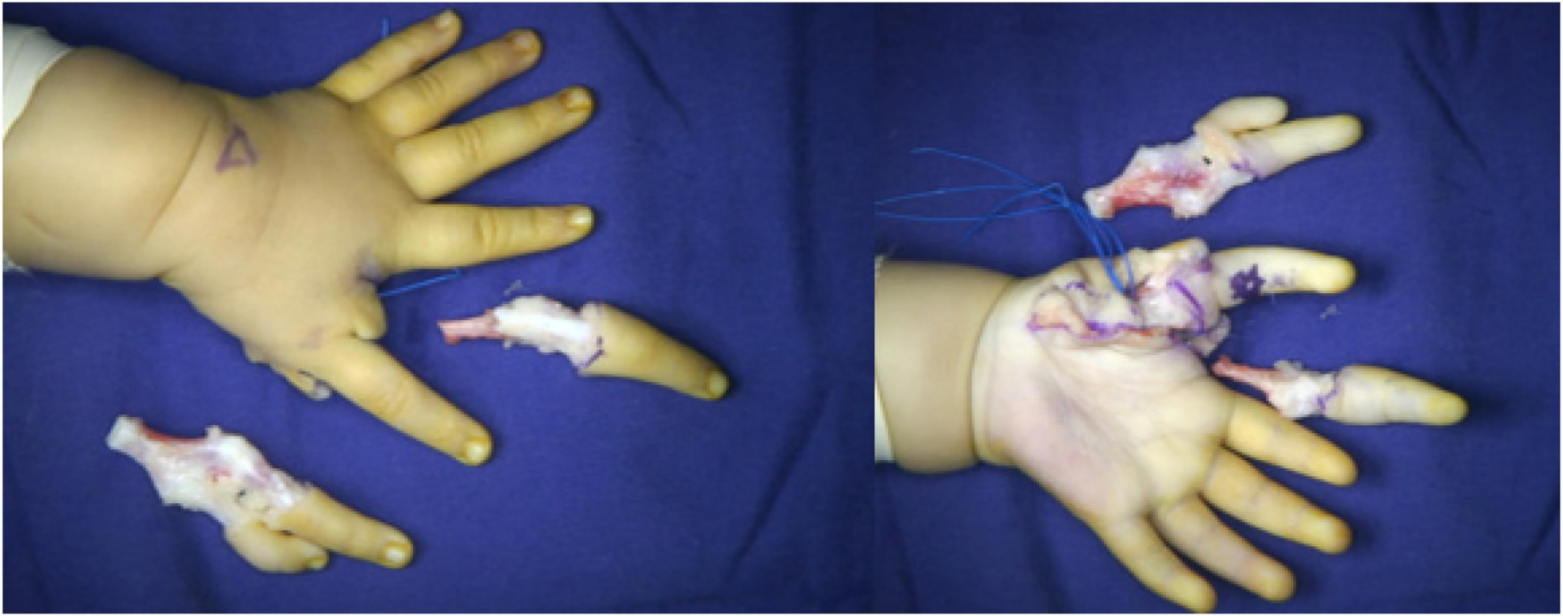
Excision of the mirror ring finger and little finger.

**Figure 9:**
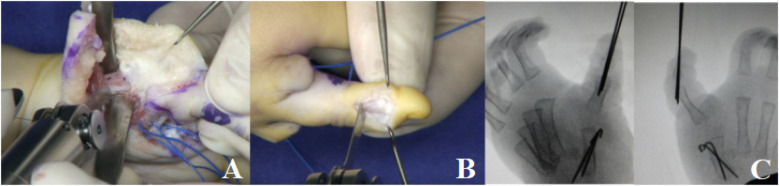
**(A)** The metacarpal was shortened by osteotomy. **(B)** The mirror middle finger was pollicized. **(C)** Intraoperative C-arm fluoroscopy confirmed satisfactory fracture reduction and alignment.

**Figure 10:**
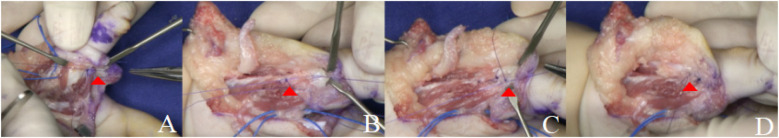
**(A)** The lumbrical muscle from the mirror index finger. (red triangle) **(B,C)** Intrinsic muscles from the mirror ring and little fingers.(red triangle) **(D)** The extensor tendons of the mirror ring and little fingers.(red triangle).

**Figure 11:**
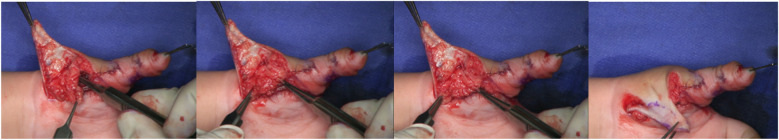
The subcutaneous tissue flap was folded and used to augment the thenar region.

Two surgical incisions were designed. The first was a circumferential incision at the level of the proximal phalanx of the mirror index finger, which extended proximally along the thenar crease on the volar aspect to the base of the palm. The second incision extended from the volar aspect of the proximal phalanx of the mirror index finger toward the radial border of the palm. The mirror middle finger was maintained. The radial palmar tissue flap was retained. The mirror index finger retained dorsal skin coverage of the first web space (Figure 5). Through the first incision, a palmar aponeurotic-like structure tissue was identified and excised beneath the subcutaneous layer. The mirror index finger and the ulnar index finger shared a common digital nerve and artery bundle. A similar neurovascular bundle was found between the four radial fingers; however, the neurovascular bundle was absent on the ulnar side of the little finger. The flexor tendons of the mirror index and middle fingers were well developed, and the lumbrical muscle attachment was visible on the palmar side. Thenar muscle structures were observed, with attachments spanning from the metacarpal of the ulnar index finger to that of the mirror middle finger (Figure 6). During the volar dissection, the mirror index finger was meticulously dissected and mobilized down to the level of the metacarpal base. The metacarpal was then transected using an oscillating saw. Following lidocaine blockade, the bilateral neurovascular bundles were divided, with hemostasis achieved using bipolar electrocautery. The lumbrical muscle was detached from its insertion point and set aside for potential use. Both the extensor and flexor tendons were transected at the level of the metacarpophalangeal joint. The mirror index finger was subsequently excised in its entirety, with preservation of the metacarpal base. (Figure 7). The articular capsule between the fourth and fifth metacarpals was incised, and the mirror ring and little fingers were completely removed, preserving intrinsic hand muscles and extensor tendons (Figure 8). Based on preoperative radiographic measurements, the metacarpal of the mirror middle finger was 7 mm longer than that of the right thumb. At the midshaft level of this metacarpal, an osteotomy was performed to resect a 5 mm segment using an oscillating saw, followed by cross-fixation with two 0.8 mm titanium Kirschner wires. The distal phalanx of the right thumb measured 10 mm in length, whereas the combined length of the middle and distal phalanges of the mirror middle finger was 21 mm. A 1.5 cm longitudinal incision was made on the medial aspect of the finger's distal interphalangeal (DIP) joint. The distal end of the middle phalanx and the base of the distal phalanx were exposed, and a 10 mm osteotomy segment (including the DIP joint surface) was removed. The DIP joint was fused and the phalanx shortened by cross-fixation with two 0.8 mm titanium Kirschner wires. This resulted in a digital contour more closely resembling the two-segment morphology of a thumb. Intraoperative C-arm fluoroscopy confirmed satisfactory fracture reduction and alignment (Figure 9). The preserved lumbrical muscle from the mirror index finger was sutured to the medial aspect of the metacarpophalangeal (MCP) joint of the neothumb to augment adduction. The intrinsic muscles of the mirror ring and little fingers were transferred to the lateral aspect of the MCP joint of the neothumb to reconstruct and reinforce abduction. Additionally, the extensor tendons of the mirror ring and little fingers were coapted to the extensor tendon of the neothumb to enhance extension (Figure 10). The preserved radial-sided skin flap of the palm was advanced volarly. This revealed significant skin redundancy and thenar eminence hollowing. The excess skin was resected while preserving the superficial fascial and adipose layers. The subcutaneous tissue flap was then folded and used to augment the thenar region. The local skin flap was trimmed and transposed volarly, followed by wound closure (Figure 11).

**Figure 12:**
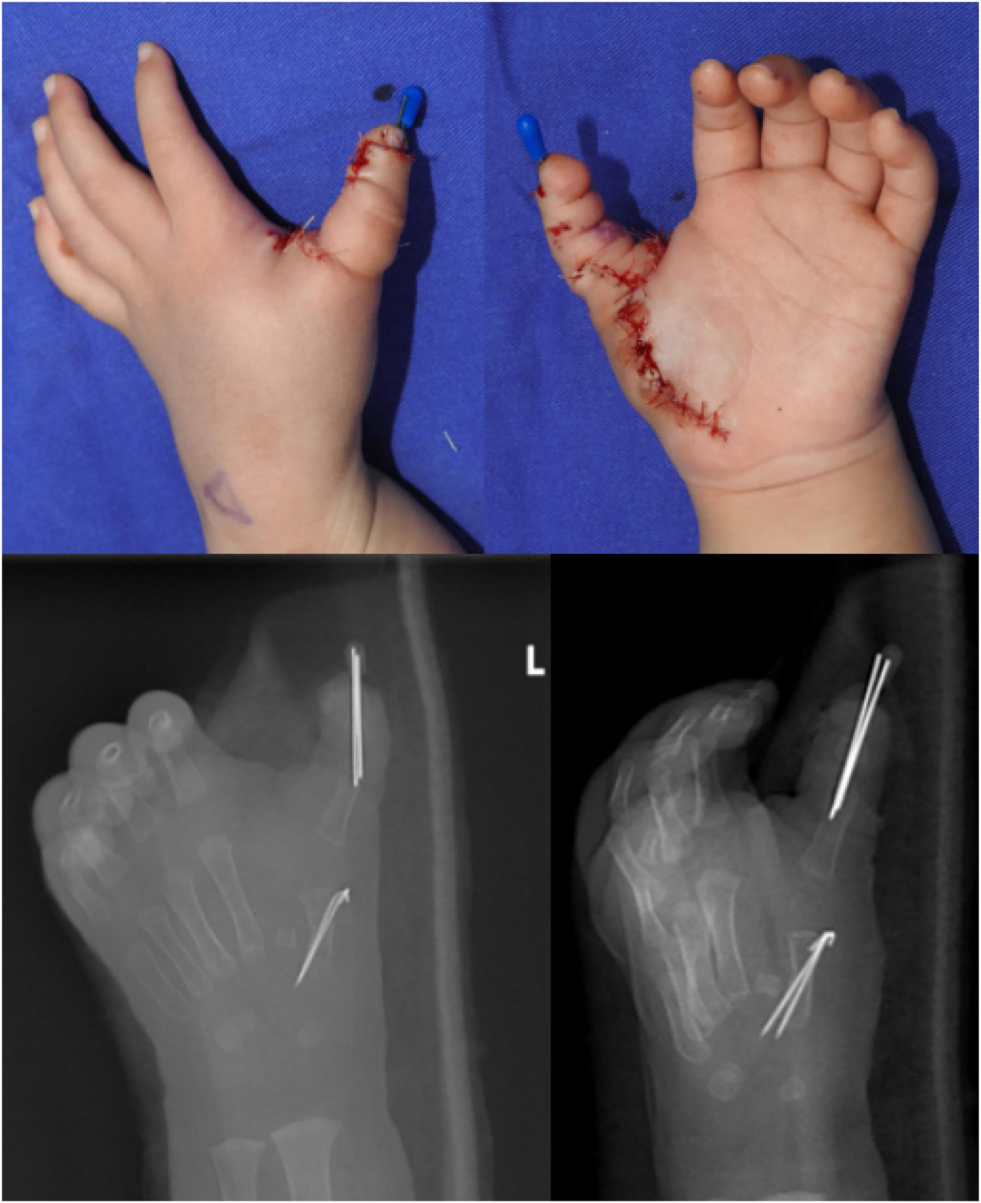
External view and orthogonal and oblique radiographs of the left hand after the operation.

Postoperative appearance and anatomical structure of the left hand showed significant improvement (Figure 12). Postoperatively, the reconstructed thumb was fixed with Kirschner wires for six weeks, with concomitant splinting for the affected digits. The K-wires were removed at the 6-week mark, upon which a structured regimen of play-based therapy and active/passive range of motion exercises was initiated to prevent joint stiffness.

**Figure 13:**
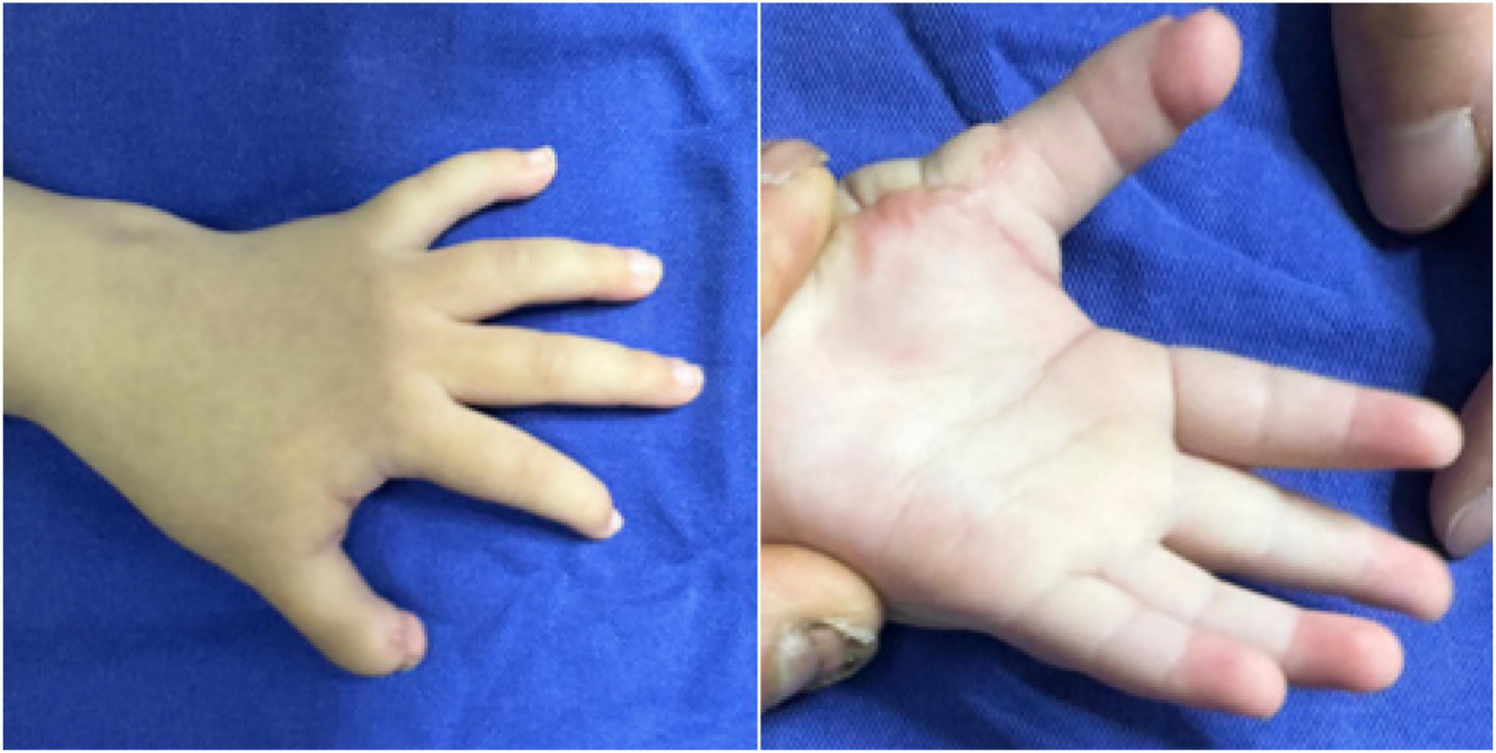
External view of the left hand at the 5-month follow-up period.

**Figure 14:**
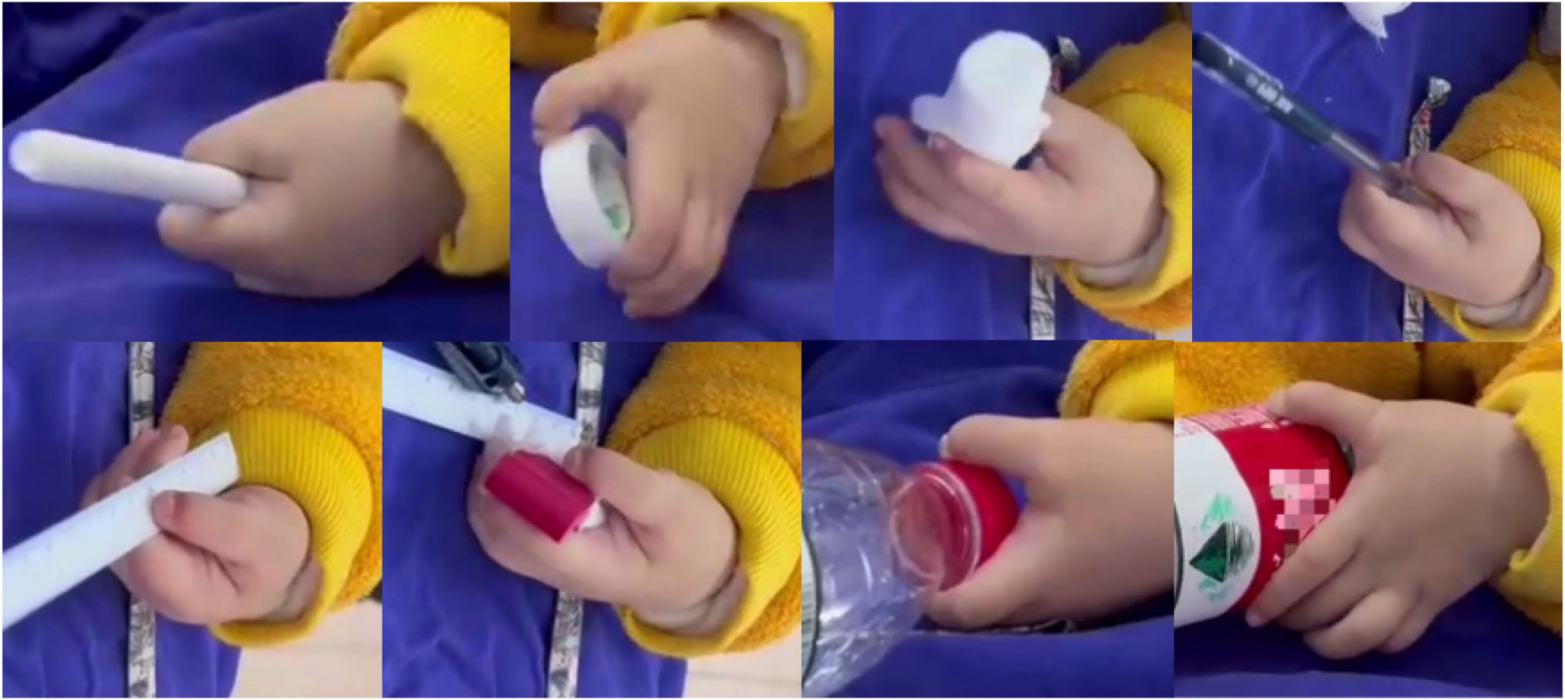
At the 5-month postoperative follow-up, the appearance and active grasping function of the left hand were significantly improved.

**Figure 15:**
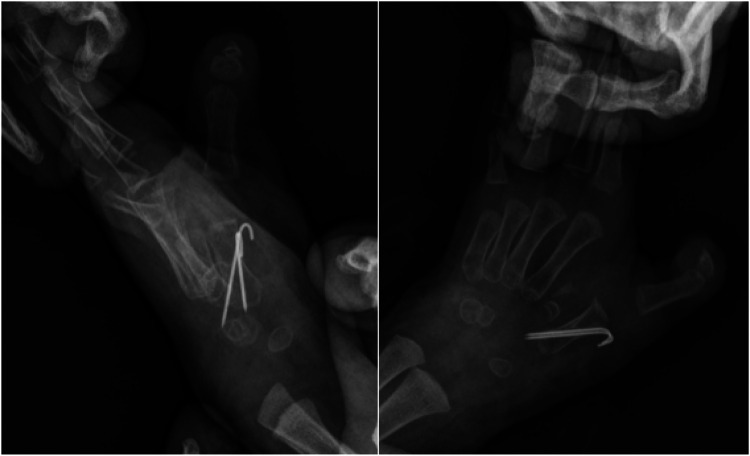
Postoperative 5-month anteroposterior and lateral radiographs of the left hand.

At the 5-month follow-up, the patient's left hand exhibited significant improvements in both cosmetic appearance and functional use. Radiographic examination confirmed satisfactory bone alignment (Figures 13–15, video 1-2 (Supplementary materials)). However, a hypertrophic scar was noted at the surgical site. To manage this and prevent contracture, a protocol of daytime exercises combined with nighttime splinting in a functional position was prescribed.

**Figure 16:**
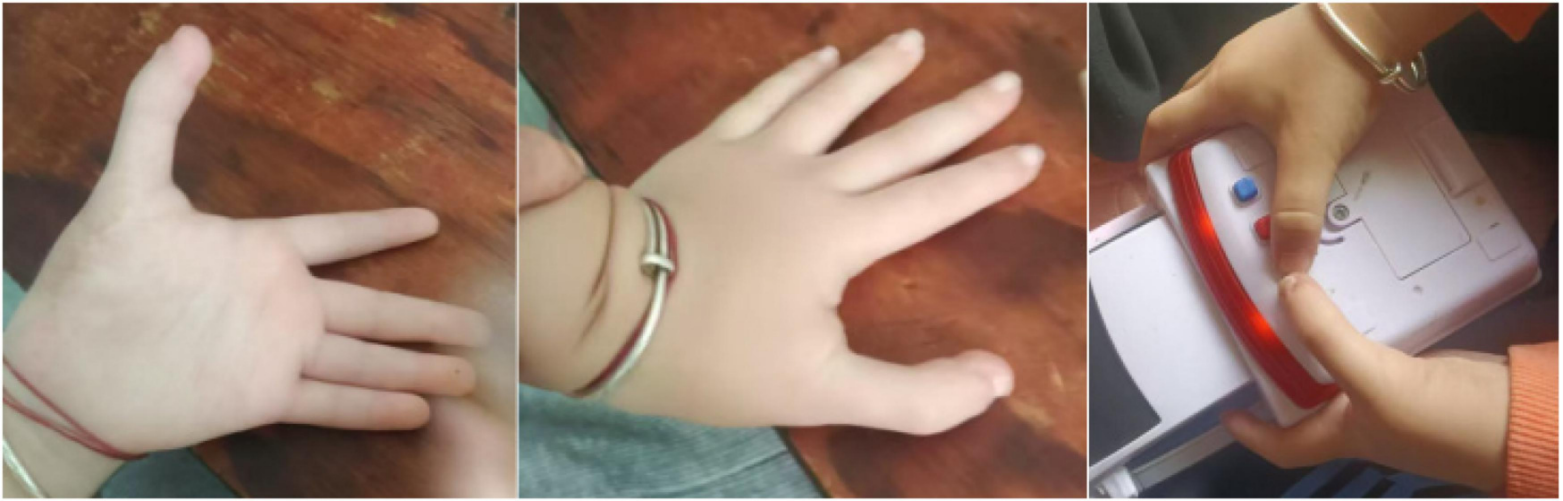
At the 23-month postoperative follow-up, the left palm showed no signs of hypertrophic scarring or contracture. Similarly, no significant scarring was observed on the dorsal aspect of the hand.

The patient was followed longitudinally, with a final assessment at 23 months postoperatively. At this stage, the palmar scar had matured into a stable structure with no signs of contracture (Figure 16, video 3 (Supplementary materials)). Longitudinal observation revealed that the reconstructed left thumb, which initially presented with a near-normal morphology compared to the contralateral side, gradually developed a characteristically slender contour during growth. Most importantly, the operated digits maintained full function for activities of daily living, with no evidence of recurrence or functional limitation.

## 3 Discussion

Non-syndromic mirror hand deformity is considered a sporadic congenital disorder, with no specific causative gene identified to date. Genetic testing of the patient in the present study and his parents revealed no significant abnormalities.

Ulnar duplication is an extremely rare condition. Typically, the duplicated ulna on the radial side is hypoplastic and short, causing radial deviation of the hand and shortening of the affected limb compared with the contralateral side (7). Consistently, our patient's left forearm was approximately 1 cm shorter than the right.

Skeletal malformations in mirror hand deformity are often accompanied by arterial and neural variations, such as absence of the radial artery, duplication of the ulnar artery, shortening of the radial nerve, and duplication of the ulnar nerve (sometimes with collaterals to the median nerve) (8). In the present case, the mirror index finger and the ulnar index finger shared a common digital nerve and artery bundle. Additionally, a nerve bundle accompanied the common digital artery between the four radial fingers, while the neurovascular bundle on the ulnar side of the little finger was absent.

In terms of intrinsic hand musculature, the radial interosseous muscles often demonstrate more severe dysplasia than the ulnar muscles, and the radial thenar muscles are frequently hypoplastic or absent (9, 10). In this patient, the flexor tendons of the mirror index and mirror middle fingers were well developed, and lumbrical muscle attachments were clearly visible on the palmar side. A thenar muscle structure was observed, spanning from the metacarpal of the ulnar index finger to that of the mirror middle finger.

Numerous surgical protocols have been established for the treatment of mirror hand deformity. 1. Pollicization of a radial digit (7, 8) requires the selection of the most dominant finger, utilizing the skin and subcutaneous soft tissue of the excised digit to reconstruct the first web space. 2. Simple excision of the radial polydactyly (9), over time and with adaptation, may allow the most radial digit to acquire some degree of opposition and pulp-to-pulp pinch function. 3. Index finger pollicization combined with excision of the supernumerary digit (10) is indicated in cases of congenital thumb hypoplasia, effectively improving both the appearance and function of the thumb. 4. The two-stage rotational osteotomy of the preserved first digit2, while necessitating a second surgical procedure, significantly reduces the risk of vascular injury. However, the existing literature lacks focused investigation into how postoperative scar appearance affects psychological development and social well-being. We developed a novel palmar-based incision design, providing excellent intraoperative visualization and anatomy preservation. The lumbrical muscle from the mirror index finger, intrinsic muscles, and extensor tendons from the mirror ring and little fingers were preserved and repositioned to restore opposition, adduction, and extension functions of the reconstructed thumb. Additionally, this approach allowed for concealed postoperative scarring. This approach significantly improves both thumb appearance and function while creating a well-concealed scar that is virtually undetectable from the dorsal aspect. The superior cosmetic outcome minimizes potential psychological impacts and enhances social comfort for the child, leading to higher parental satisfaction.

Mirror hand polydactyly differs from common polydactyly: the thumb is absent and replaced by two or more fingers functioning collectively to provide opposition (15). We emphasize the importance of reducing redundant digits, reconstructing a functional and anatomically appropriate thumb, and carefully selecting the most dominant finger for pollicization (4). In our patient, the mirror middle finger and ulnar palm performed effective palmar opposition, leading us to select the mirror middle finger as the dominant digit for thumb reconstruction. Osteotomy was performed on this digit to replicate normal thumb morphology. The mirror ring, little, and index fingers were excised, while the base of the second metacarpal was preserved to maintain carpometacarpal joint stability and provide a bony structure for widening the first web space.

Mirror hand deformity may recur or present with functional and structural challenges after the first surgical reconstruction, underscoring the need for long-term follow-up. While surgical correction can be effective at any age, early intervention is preferred (16). The optimal age for hand reconstruction is before 2 years of age (11). Ultimately, the most significant outcome is the full functional integration of the reconstructed hand in activities of daily living over nearly two years of follow-up. The absence of recurrence validates the efficacy of the surgical strategy in not only addressing the present deformity but also in ensuring lasting functionality.

A particularly noteworthy finding was the evolution of the reconstructed thumb's morphology. The initial minimal difference gave way to a characteristically slender contour over time. This suggests that the growth potential of the preserved skeletal elements may follow its own inherent pattern, differing from that of a normal thumb. This observation has important implications for preoperative family counseling regarding long-term cosmetic expectations.

The development of a hypertrophic scar, while a known complication in pediatric hand surgery, was effectively managed with a conservative regimen of therapy and splinting. Its subsequent maturation into a non-contractile scar by the 23-month follow-up underscores the importance of persistent postoperative scar management in young children.

The family expressed satisfaction with the surgical outcome. Owing to the significant restriction of wrist and elbow joint movements caused by the duplicated ulna, the patient exhibited decreased use of the left hand. The family has expressed interest in early surgical intervention to improve forearm and elbow function. Effective management of mirror hand deformity requires addressing the entire upper limb, not just the hand and wrist (17). Therefore, considering rehabilitation needs and psychosocial factors related to preschool age, the patient has been scheduled for a follow-up appointment to evaluate bilateral ulnar procedures, with concurrent removal of the first metacarpal Kirschner wire to mitigate potential impacts on its longitudinal growth.

For future cases, we intend to incorporate 3D printing technology to enhance anatomical accuracy and functional outcomes in upper limb reconstruction (18). We will continue regular follow-up and look forward to reporting outcomes after subsequent procedures, contributing insights for the treatment of similar congenital upper limb anomalies.

This study highlights the preliminary technical feasibility and potential application value of the new surgical technique. However, given the limitations inherent in a single case report, further cases and long-term follow-up are required to validate its efficacy.

The study protocol received ethical approval from the institutional review board [Reference No: 2025(L)005]. The child's legal guardian provided written informed consent for the publication of all medical images. The consented images and data have been included in the supplementary materials.

## Data Availability

The original contributions presented in the study are included in the article/Supplementary Material, further inquiries can be directed to the corresponding author.

## Supplementary material

Supplementary Video 1 4 months after surgery.

Supplementary Video 2 5 months after surgery.

Supplementary Video 3 23 months after surgery.
